# Ecology and Biogenesis of Functional Amyloids in *Pseudomonas*

**DOI:** 10.1016/j.jmb.2018.05.004

**Published:** 2018-10-12

**Authors:** Sarah L. Rouse, Stephen J. Matthews, Morten S. Dueholm

**Affiliations:** 1 Department of Life Sciences, Imperial College London, South Kensington Campus, London, SW72AZ, UK; 2 Center for Microbial Communities, Department of Chemistry and Bioscience, Aalborg University, Aalborg, Denmark

**Keywords:** Fap, functional amyloid in *Pseudomonas*, EPS, extracellular polymeric substances, PGPR, plant growth promoting rhizobacteria, QS, quorum sensing, PQS, 2-heptyl-3-hydroxy- 4(1*H*)-quinolone, 3-oxo-C12-HSL, *N*-(3-oxododecanoyl)-l-homoserine lactone, Fap, amyloid, diversity, structure, sequence covariance analysis

## Abstract

Functional amyloids can be found in the extracellular matrix produced by many bacteria during biofilm growth. They mediate the initial attachment of bacteria to surfaces and provide stability and functionality to mature biofilms. Efficient amyloid biogenesis requires a highly coordinated system of amyloid subunits, molecular chaperones and transport systems. The functional amyloid of *Pseudomonas* (Fap) represents such a system. Here, we review the phylogenetic diversification of the Fap system, its potential ecological role and the dedicated machinery required for Fap biogenesis, with a particular focus on the amyloid exporter FapF, the structure of which has been recently resolved. We also present a sequence covariance-based *in silico* model of the FapC fiber-forming subunit. Finally, we highlight key questions that remain unanswered and we believe deserve further attention by the scientific community.

## Highlights

• New evolutionary analysis identifies the Fap system in 25 additional genera.
• The list of known Fap protein homologs is greatly expanded for *Pseudomonas*.
• Recent structural and mechanistic insight into the Fap amyloid secretion system
• Structural model for FapC in the amyloid state derived from sequence covariance

## Graphical Abstract

**Figure:**
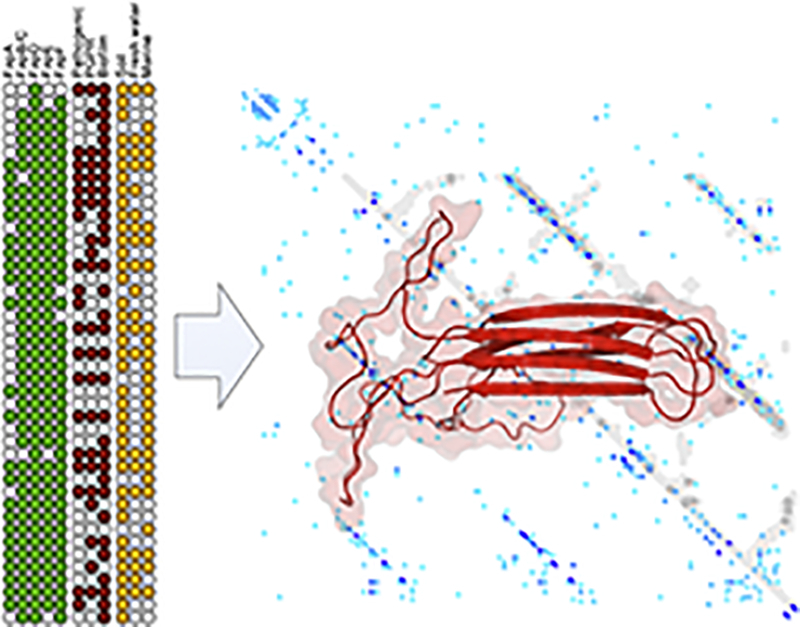

## Introduction

In nature, bacteria rarely live as isolated individual cells. Instead, they form highly organized communities embedded in three-dimensional scaffolds of hydrated extracellular polymeric substances (EPS). This is referred to as the biofilm lifestyle [1]. Bacterial biofilms can lead to chronic infections due to a markedly increased resistance to antibiotics [2], [3], and in water distribution systems, biofilms cause fouling and biodeterioration [4]. However, biofilms may also serve useful purposes, for example, in biofilters, where the metabolism of surface-attached bacterial biofilms is harnessed to pollutant degradation [5]. Thus, while microbial biofilms can be of great benefit for humans, they can also be a significant nuisance, causing expense or life-threatening diseases. To control biofilm formation, it is essential that we understand the properties and ecological importance of the individual EPS components.

Functional amyloids are found in biofilms from diverse habitats and represent a remarkable class of EPS molecules [6], [7], [8]. These highly ordered protein fibrils are defined by a characteristic cross-β-sheet quaternary structure and an ability to self-assemble from their monomeric counterparts in a nucleation-dependent process [9]. The amyloid structure is often associated with high structural stability toward denaturation and proteolytic degradation [10]. Amyloids are therefore ideal scaffolds for biofilm assembly [6], [7], [8]. However, they may also have more specialized functions in mediating bacteria–host interaction [11] and binding of quorum sensing (QS) molecules [12].

**Fig. 1:**
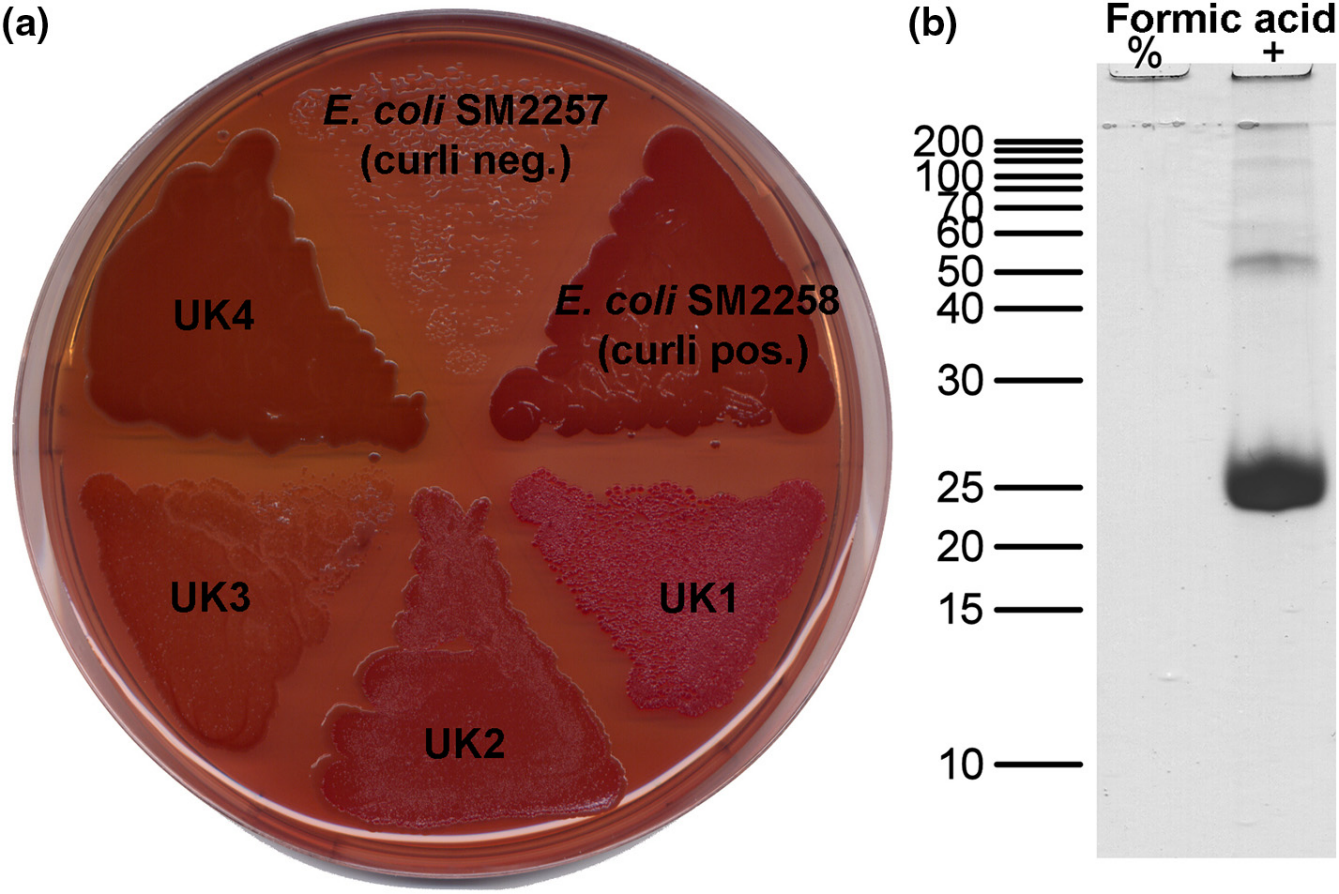
Brute force screening for novel functional amyloid systems. (a) Individual bacterial isolates were spread on Congo red agar plates [14] and evaluated for amyloid production based on their ability to bind the amyloid-specific dye Congo red. Curli-negative and -positive *E. coli* strains were used as a control. Unknown bacteria (UK1–4) number 4 was later identified as *Pseudomonas* sp. UK4. (b) Congo red-positive strains were subjected to amyloid purification and analyzed by SDS-PAGE with or without prior treatment with concentrated formic acid, known to depolymerize functional amyloids. The gel shows the analysis for *Pseudomonas* sp. UK4. The thick protein band at 25 kDa corresponds to the major amyloid subunit FapC.

A handful of evolutionarily unrelated functional amyloid systems have been described so far. The best characterized of these is the curli system, which was first identified in *Escherichia coli* [13] and *Salmonella* species [14] and later shown to be present in more than 40 bacterial genera belonging to at least four bacterial phyla [15]. The discovery of functional amyloids in *E. coli* inspired a hunt for novel amyloid systems in other species, which is highlighted by the recent discovery of a functional amyloid system in *Pseudomonas* (Fap) (Fig. 1) [16], [17]. Although the Fap system displays many similarities to the curli system, the two are not evolutionary related, and recent studies into the biogenesis have revealed some intriguing differences. Here we will provide a critical overview of the functional amyloids in *Pseudomonas* with focus on the evolution, the ecological importance and the dedicated molecular machinery used to ensure spatial and temporal control the amyloid biogenesis.

## Evolution of the Fap system

The major subunit of the amyloid fibril (FapC) is synthesized from a six-gene operon (*fapABCDEF*), which also encodes the necessary auxiliary components required for Fap biogenesis [16]. The *fap* operon was originally identified in several *Pseudomonas* species, including strains of the opportunistic pathogen *Pseudomonas aeruginosa*, the plant growth promoting rhizobacteria (PGPR) *P. protogenes* and the bioremediation-related *P. putida* [18]. However, a subsequent evolutionary study revealed that the *fap* operon was not restricted to the *Pseudomonas*, but could, in fact, be found in 14 additional genera all belonging to the phylum Proteobacteria [17]. Comparative analysis of phylogenetic trees based on Fap proteins and the traditional taxonomic marker, the 16S rRNA gene, demonstrated that horizontal gene transfer represented a minor player in the evolution of the Fap system [17].

**Fig. 2:**
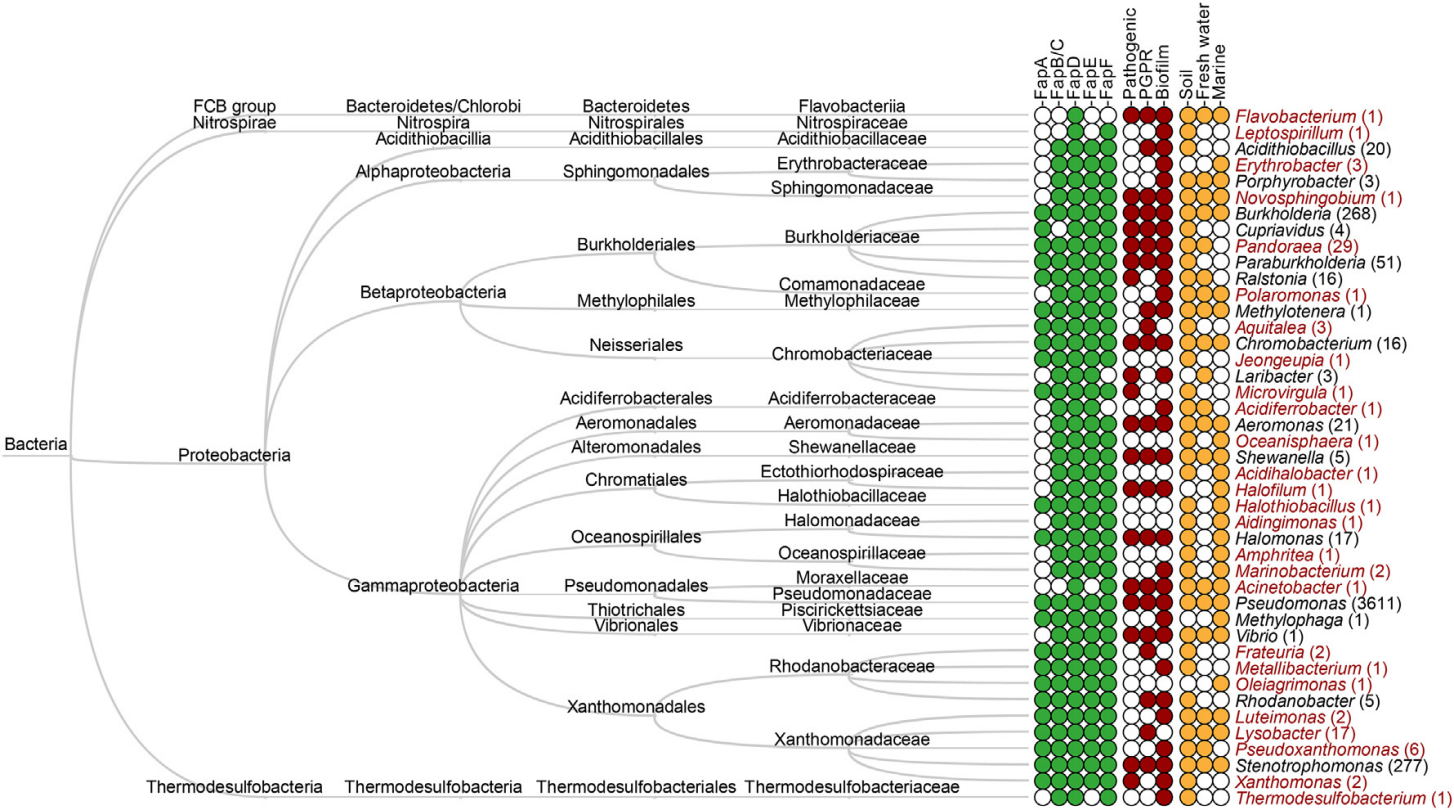
Phylogenetic distribution of the Fap system. Fap protein homologs were identified in the non-redundant bacterial RefSeq protein database v. 83 using the previously described profile hidden Markov models [17]. NCBI's Batch Entrez was used to extract metadata for identical protein groups, and this information was used to identify genomes containing *fap*-operon homologs. Only proteins that were encoded in a *fap-*operon (< 5000 bp to second nearest *fap*-gene neighbor) with at least four *fap*-gene homologs were included in the analysis. This was done to remove false positives. The taxonomic analysis was performed based on the NCBI taxonomy and visualized using MEGAN 6.0 [19]. Numbers in parentheses indicate the number of strains containing Fap systems within each genus. Note that these numbers are highly influenced by the number of sequenced strains within each phylogenetic group and therefore do not reflect the prevalence of Fap systems within these groups. Genera highlighted in red represent new genera where the Fap systems have been identified in this study. The presence of individual Fap components and selected metadata and habitat for each genus is illustrated in the filled circles left of the genus names. PGPR, plant growth promoting rhizobacteria.

The number of genome-sequenced bacteria in the reference databases has expanded greatly since the initial evolutionary analysis carried out in 2012 due to major advances in DNA sequencing technologies. To expand our understanding of Fap evolution, we used the previous Fap profile hidden Markov models to search for additional genomes encoding the Fap system in the current version of NCBI's bacterial reference database (RefSeq v. 85) (Fig. 2 and Supplementary data 1). This revealed 25 additional genera that encode the complete Fap system and expanded the number of strains within the previously identified genera significantly, for example, 32 *versus* 3611 for *Pseudomonas*. The huge increase in the number of protein homolog provides a foundation for bioinformatic identification of functional relevant domains or key-residues within individual Fap proteins, for example, by analyses of sequence evolution using *K*a/*K*s ratios [20] or by identification of correlated mutations in the protein sequences [21]. Furthermore, we observed colocalized *fapD* and *fapF* gene homologs (< 5000 bp apart) in five additional phyla, suggesting an evolutionarily conserved interaction between the encoded proteins, FapD and FapF (Fig. S1). [21].

## Ecological importance

Fap fibrils were first discovered in *Pseudomonas* sp. UK4, an environmental strain isolated from a biofilm growing on a glass slide submerged in a drinking water reservoir [16]. Accordingly, the amyloids were proposed to act as adhesins or structural components of biofilms. This function was later confirmed using recombinant mutants of several *Pseudomonas* species, which upon overexpression of the *fap* operon produced highly aggregative phenotypes that formed unusually strong biofilms [18]. Atomic force microscopy with colloidal probes was used to measure the mechanical properties of biofilms produced by wild type and amyloid overexpressing mutants [22]. This revealed that the amyloid expression greatly increased the overall hydrophobicity and stiffness (> 20-fold) of the biofilms. The enhanced stiffness is likely caused by the unique mechanical properties of amyloid fibrils, which act as a rigid scaffold for the more elastic biofilm matrix components such as eDNA and polysaccharides [22]. The amyloids were also found to fortify and increase the hydrophobicity of individual cells and thereby increase their resistance to dehydration [22]. This provides a competitive advantage for bacteria that encounter environments where there are frequent periods of low water activity such as in soils [22]. This revealed that the amyloid expression greatly increased the overall hydrophobicity and stiffness (> 20-fold) of the biofilms. The enhanced stiffness is likely caused by the unique mechanical properties of amyloid fibrils, which act as a rigid scaffold for the more elastic biofilm matrix components such as eDNA and polysaccharides. The amyloids were also found to fortify and increase the hydrophobicity of individual cells and thereby increase their resistance to dehydration. This provides a competitive advantage for bacteria that encounter environments where there are frequent periods of low water activity such as in soils.

Although the Fap fibrils have a clear use as structural elements in the biofilm, they also have additional specialized functions. These include an ability to bind QS molecules and redox mediators [12]. Measurements of binding kinetics between *P. aeruginosa* FapC fibrils and the QS molecules 2-heptyl-3-hydroxy- 4(1*H*)-quinolone (PQS) and *N*-(3-oxododecanoyl)-l-homoserine lactone (3-oxo-C12-HSL), as well as the redox mediator pyocyanin, revealed a dynamic association [12]. This facilitates the bioavailability of the molecules inside the biofilm while ensuring a high concentration in the vicinity of the producing cells, even in turbulent conditions. Binding of the redox mediator pyocyanin was also shown to alter the conductive properties of the Fap fibers [12]. This may have direct implications for the transfer of electrons through biofilms. Homologous expression of the *fap*-operon, which does not encode any regulatory proteins, was previously shown to cause profound changes to the proteome of *P. aeruginosa* PAO1 [23]. This supports the hypothesis that the amyloids interfere extracellular communication and regulation. These changes included the downregulation of classical virulence factors and the upregulation of biofilm-associated pathways including activation of the alginate biogenesis machinery. The result was a mucoid phenotype with properties similar to those observed for *P. aeruginosa* strains isolated from chronic infections [24]. Based on these observations, it is tempting to speculate that *P. aeruginosa* uses amyloids to establish chronic wounds or infections in patients suffering from cystic fibrosis [23]. This supports the hypothesis that the amyloids interfere extracellular communication and regulation. These changes included the downregulation of classical virulence factors and the upregulation of biofilm-associated pathways including activation of the alginate biogenesis machinery. The result was a mucoid phenotype with properties similar to those observed for *P. aeruginosa* strains isolated from chronic infections. Based on these observations, it is tempting to speculate that *P. aeruginosa* uses amyloids to establish chronic wounds or infections in patients suffering from cystic fibrosis.

There are to our knowledge no studies that have directly investigated the role of Fap fibrils in chronic infections, although indications for their involvement have been supported by different studies. A deletion mutant of *P. aeruginosa* PAO1 lacking the gene encoding the major Fap subunit, FapC, was found to be among the most attenuated strains in both a *Caenorhabditis elegans* infection model and a polymorphonuclear neutrophil leukocyte phagocytosis assay among 480 random transposon deletion mutants [25]. Transcription of the *fap* operon was also observed to be highly up-regulated in murine models of acute burn and chronic surgical wound infections when compared to standard laboratory growth conditions [26]. Finally, amyloids have been observed in murine-based corneal infection and implant models [27]. The potential role of Fap fibrils in pathogenesis is intriguing as many species that cause chronic infections encode the Fap system. These include several species contributing to severe pulmonary infection in immunocompromised individuals and patients suffering from cystic fibrosis, including *P. aeruginosa* [28], *Burkholderia cepacia* [29], *B. gladioli* [30], *Pandoraea* sp. [31], *Ralstonia pickettii* [32] and *Stenotrophomonas maltophilia* [33]. If the Fap fibrils are essential for the infections, these molecules should be considered as potential targets for anti-biofilm drugs.

It is not only pathogenic strains that encode the Fap system. The Fap system may therefore also have environmental relevance. Many members of the genus *Acidithiobacillus* can oxidize mineral sulfides under acidic conditions, and these bacteria are accordingly used for bioleaching [34], [35]. Biofilm formation on the mineral sulfides enhances the oxidation process, and it would be interesting to see if Fap fibrils are used in this process [36]. Another interesting observation is the presence of the Fap system in the biofilm producing phototrophic bacteria *Erythobacter*, *Porphyrobacter* and *Novosphingobium* [37], [38]. Phototrophic biofilms are currently being exploited for applications in wastewater treatment, bioremediation, aquaculture and biohydrogen production [39]. Accordingly, it would be relevant to investigate the possible role of amyloids in these systems. Finally, it should be noted that many known root-colonizing PGPR, which belongs to the Burkholderiales, Pseudomonadales and Xanthomonadeles orders [40]. An involvement of functional amyloids in root colonization has been described for the curli producing *Enterobacter cloacae* GS1. It is therefore plausible that the Fap fibrils serve a similar purpose for some PGPR.

## Molecular insights into Fap biogenesis

Our understanding of how the Fap system functions on a molecular level hinges on resolving the atomic structures of each protein, as well as determining the interactions between them and the conformational changes that take place during biogenesis. The six Fap proteins are synthesized in the cytosol before being exported into the periplasm via the SecYEG pathway with corresponding signal sequence cleavage [41]. Three of the mature proteins, FapB, FapC and FapE are then secreted extracellularly via the outer membrane protein FapF to form, or be associated with, the amyloid fiber. The major fiber-forming subunit FapC contains three amyloid repeats. FapB is an internal homolog of FapC and has a shorter repeat pattern, and has thus been suggested to be a nucleator of FapC, analogous to CsgB in curli [42]. FapE has also been detected externally in the Fap fibers [18]. FapF is the membrane-spanning component through which FapB, FapC and FapE are exported. The roles of FapA and FapD are less clear. FapA is the only Fap protein that is not vital for fiber formation, and the absence of FapA is the most common genetic variants [17]. Deletion of *fapA* in *Pseudomonas* was shown to alter the relative fiber composition of subunits FapB and FapC. Therefore, FapA has been proposed to have a regulatory role, possibly inhibiting FapC fibrillation [18].

FapD is a structured protein, homologous to the C39-peptidase family [16]. The canonical C39 peptidases cleave proteins at a conserved GG motif. However, some subsets exist purely as a chaperone rather than processing their substrates*. In vivo* assays have indicated that the catalytic Cys38 residue in FapD is required for FapC secretion [41]. This suggests that FapD is likely to actively process one or more of the Fap proteins, perhaps in addition to possessing a chaperoning role [41], [43], [44]. The substrate of FapD is yet to be determined. However, mass spectrometric analysis of whole cells revealed peptides corresponding to the N-terminus of all Fap proteins except FapE, hinting at a possible cleavage of FapE [41].

**Fig. 3:**
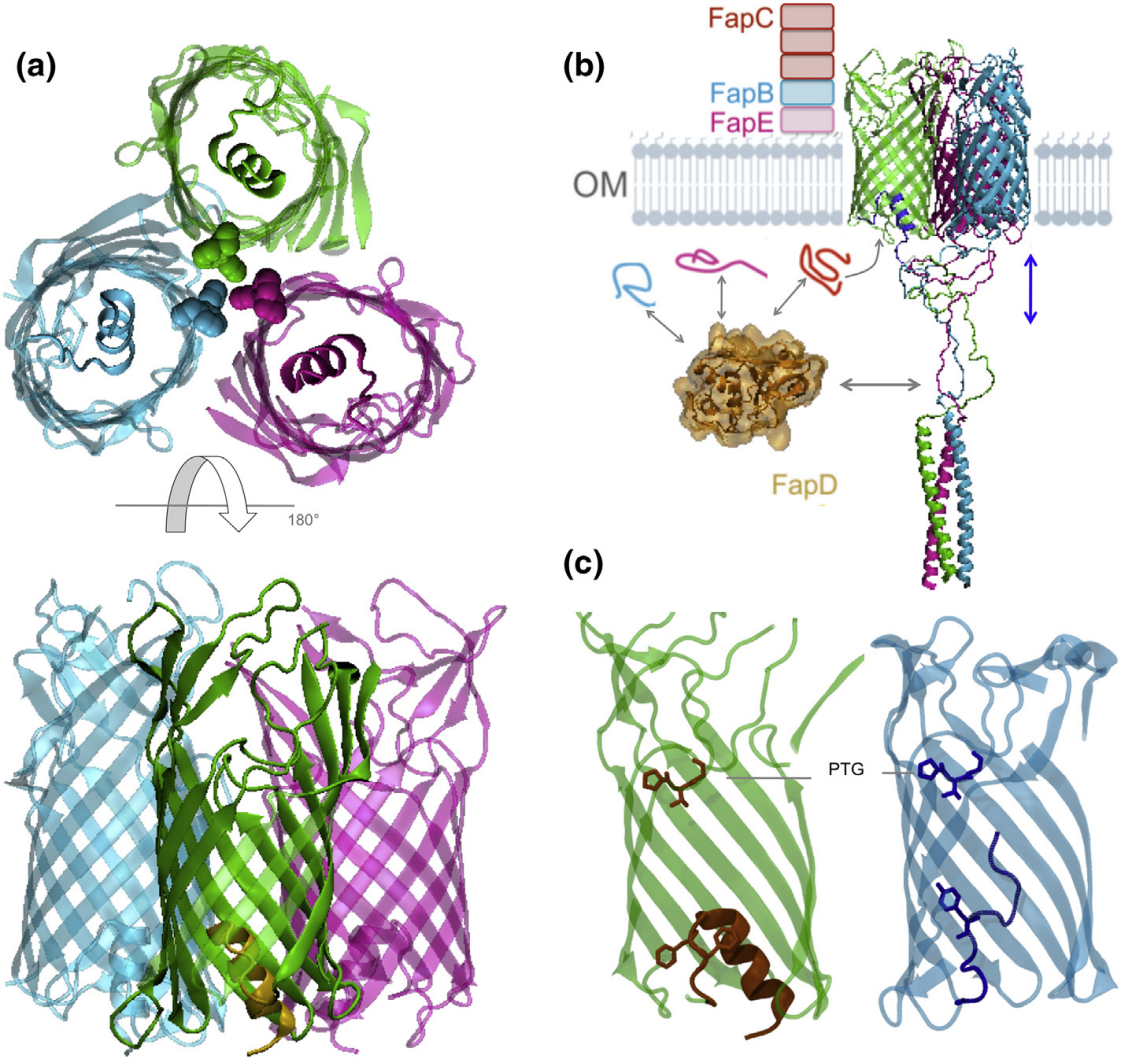
Recent structural insight into the Fap secretion system. (a) The structure of FapFβ (PDB ID 5o56) through which the Fap fiber-associated subunits FapB, FapC and FapE are exported. Top panel: the view from the periplasm highlighting the helical plugs constricting each pore, and the trimeric packing interface of Phe residues. Lower panel: side view of FapFβ from the plane of the membrane. (b) The periplasmic domain of FapF was shown to be a trimeric coiled coil, residing in the periplasm. FapB, FapC and FapE are secreted through FapF which must require motion of the helix plug (blue arrow). FapD (brown) is likely to regulate the secretion by processing one or more Fap proteins. (c) Comparison to the Pput2725 transporter (PDB ID 4rl8) from the COG4313 hydrophobic substrate uptake family showing a conserved PTG motif (shown in stick representation) proposed to form a lateral substrate gate at the extracellular side of the barrels. The constriction of each channel is shown. The plug of FapF folds back out and forms a longer N-terminal domain extending from the helix, whereas the complete Pput2725 N-terminus is shown threading up into the barrel. Phe102/103 of the FapF and Tyr8 of the Pput2725 “plugs” are shown.

The first structural insight into the Fap family of proteins was achieved by the recent crystal structure of the membrane-spanning domain of FapF, FapFβ [41], [45]. This provided the first molecular-level insight into the control of amyloid formation in *Pseudomonas*. FapFβ was shown to form a trimeric, 12-stranded β-barrel, which is constricted by a 12-residue helix on the periplasmic side (Fig. 3a).

FapF is often annotated as autotransporter family based on it having a 12-stranded β-barrel containing the “mortise-tenon” motif [46], [47]. However, recent work highlights that FapF is not a conventional autotransporter protein. Autotransporters possess an N-terminal passenger domain that is transported across the outer membrane, either during or after barrel membrane insertion. A subsequent self-catalyzed cleavage can also take place to release the passenger domain [48]. In the case of FapF, the N-terminal domain is not secreted but instead forms a stable trimeric coiled-coil in the periplasm (Fig. 3b), and this links to a helical plug within the barrel lumen. This periplasmic domain was shown to be vital for FapC secretion, as well as driving the trimerization of full-length FapF [41]. To date, no direct evidence for processing of this N-terminal domain has been observed, and instead, the FapF coiled-coil domain has been proposed to play a role in regulating the gating of the helical plug, thereby regulating the secretion of the Fap components (Fig. 3b).

A comparison of FapF to the structure of the COG4313 family of outer-membrane channel Pput2725 from *P. putida* F1 revealed clear structural similarities (Fig. 3c) [49]. COG4313 proteins contribute to the uptake of hydrophobic molecules. For example, the TcpY protein from *Cupriavidis necator* has been suggested to function as an uptake channel for trichlorophenol and the SphA channel from *P. aeruginosa* was implicated in sphingomyelin uptake [50]. COG4313 proteins also possess a conserved PTG motif and this has been suggested to form a lateral gate for the release of these substrates. The presence of associated C39 peptidases has not been reported in operons possessing a COG4313-like outer membrane pore; therefore, it is conceivable that Fap relies on a “hijacked” form of these uptake channels, with FapD regulating the export of polypeptides. Furthermore, it is conceivable that the Fap systems respond to the presence of certain hydrophobic, quorum sensing or lipid molecules via an interaction with the FapF transmembrane barrel.

## Model of FapC derived from sequence covariation

The structure of FapF provides the first structural insights into Fap biogenesis. However, elucidating the three-dimensional structures of the remaining Fap proteins, and their complexes will provide the next step forward in our mechanistic understanding of this system. FapC is a key target, not only because it is the main amyloid-forming component and therefore possesses many of the functional properties of Fap fibers, but also because it provides the inspiration for new biosynthetic materials with unique properties, as has been demonstrated for the curli system [51], [52], [53].

Fap and curli fibers have shared features. FapC fiber formation can be inhibited *in vitro* by the curli inhibitor CsgC [54]. The morphology of the fibers is similar, and a conserved Q/N-X_10_-Q/N repeat exists within CsgA, FapB and FapC sequences [55]. Significant differences also exist. The three 37-amino acid repeats of FapC are longer than those found in CsgA, which are ~ 22 residues in length, and they are connected by much larger and more variable inter-repeat regions [16], [18]. These additional regions may either form distinct domains outside the amyloid fibril core or insert to form additional strands within it. Perhaps the most significant difference is evident in membrane proteins through which they are exported. The curli transporter CsgG forms a large single, open pore through the membrane [56], [57], while FapF forms three gated channels [41]. CsgA is targeted for export through CsgG by an N-terminal peptide (N22), but no equivalent targeting sequence has been found for FapC.

**Fig. 4:**
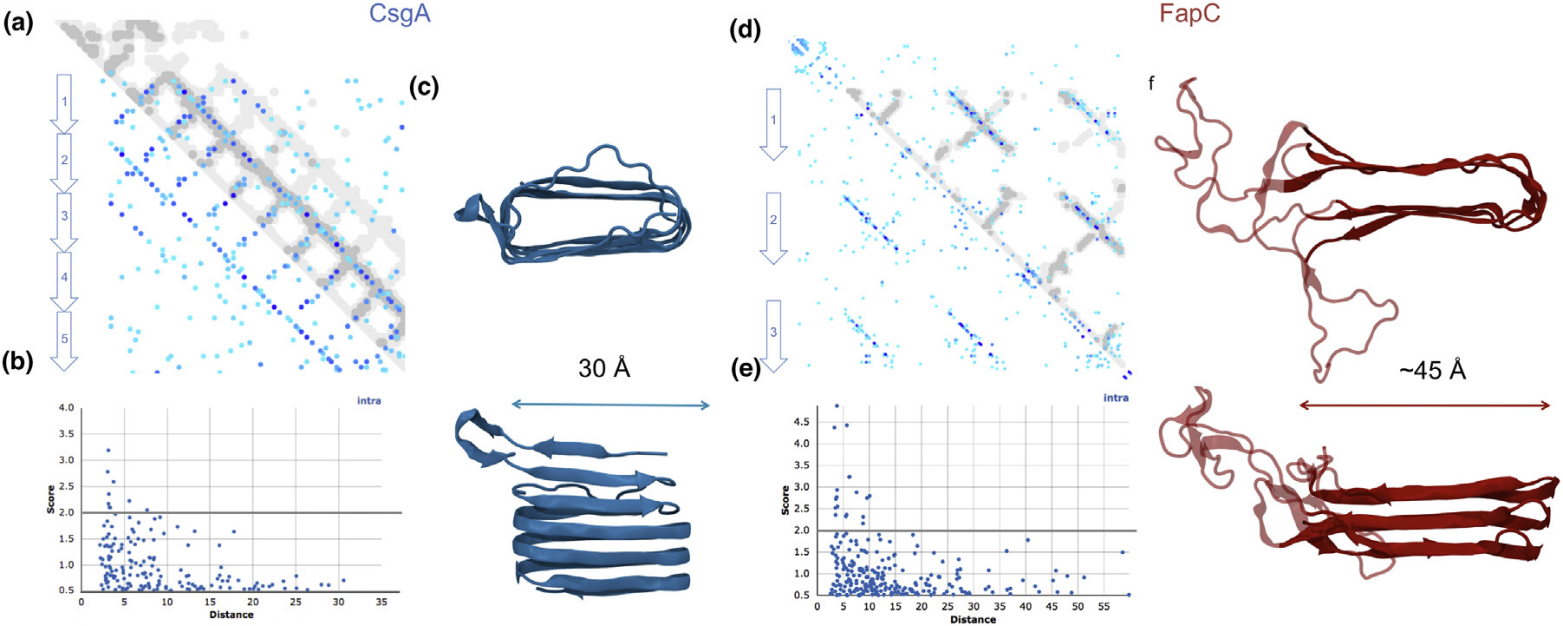
Models of the Fap and curli fibers computed using distance restraints generated from sequence covariance data. (a) Plot of predicted contacts between CsgA residues calculated using GREMLIN. The amyloid repeats of CsgA are indicated by arrows. The darker and larger the blue dots, the higher coevolution strength. The distances calculated from the coordinate file are mapped on in gray. (b) The fit to the covariance data in panel a is shown as contact score versus distance within the coordinate file. (c) Model of CsgA from Tian *et al*. [63]. (d) Predicted contacts for FapC with model overlaid, as panel a. (e) Fit of the FapC coordinates to the covariance data. (f) Model of the FapC core. Covariance data for *Pseudomonas* sp. UK4 FapC was generated using an alignment (MUSCLE v.3.8.425 with standard settings and 16 iterations [64]) of the identified FapBC homologs as an input for GREMLIN with residues 38–214 shown in the model. Larger plots are shown in the Supporting Information Figs. S2 and S3.

The major fibril component FapC is perhaps the most challenging structural target of the Fap protein family and will likely require a multidisciplinary approach to be solved. Solving the experimental structures of amyloid fibers remains a challenging prospect, as only a few structures have been solved to date [58], [59]. The recent and dramatic increase in genomic data combined with advances in sequence covariance algorithms provides a valuable opportunity to gain early structural insight into the Fap amyloid [21], [60], [61], [62]. This approach has been recently exploited for CsgA (Fig. 4d), in which an *in silico* model was generated using a combination of sequence covariance-derived restraints and NMR restraints [63].

Sequence covariance methods were not previously applicable to FapC due to the number of sequences required to produce a meaningful signal. With the new alignments described here, we have been able to identify covarying residues for FapC, using PSICOV [65], EVcoupling [61] and GREMLIN [62]. All three methods were consistent in identifying the same top “hits,” which represent pairs of residues that are predicted to be in close contact. For FapC, all the top hits correspond to predicted direct contacts between the three sets of repeat residues (Fig. 4d) and suggest that the inter-repeat regions of FapC do not insert within the amyloid core and instead are appendages to the fiber. We next generated a model of the core repeat region using idealized parallel beta strands as a template (described in Fig. S4) for the core of the FapC fiber (Fig. 4f). We tested both a two-sided and three-sided beta helix arrangement, which both fulfilled the covariance-derived contacts. Only the two-sided model remained stable in molecular simulations, both in monomeric form and within a stacked trimeric arrangement (Fig. S4), and we therefore used this as the basis for our final model. Our FapC model fits the covariance data well, as compared to the CsgA fit (Fig. 4a, b), with all the highest scoring contacts (Gremlin score > 2.0) being fulfilled by the model (Fig. 4e). The model reveals a fiber core of approximate width of 4.5 nm, which is larger than predicted for CsgA, which is 3 nm wide. We found that FapC sequence covariance is dominated by intramolecular contacts as was also observed for CsgA and bactofilin [66]. We note that the conserved CxxC motif of FapC does not form part of the fiber core and could be anticipated to play a role in polymerization and inter-fiber stabilization. The location of CxxC next to the large linker regions in FapC appears to prohibit an intrastrand interaction. No corresponding motif exists in CsgA. This initial sequence covariance-derived model of FapC could be used to assist with future structural studies of the Fap fibers. Incorporating further experimental restraints such as solid-state NMR distance restraints would provide further refinement to this model. Furthermore, we note that Louros *et al*. [67] were able to model a two stranded curli fiber using an alternative CsgA model based on an AgfA homology model by Collinson *et al*. [68] and docking approaches. Unraveling the details of interactions between FapC molecules within the fibers could provide further molecular insight and allow further comparison between the Fap and curli functional amyloid mechanisms.

## Perspective

The updated evolutionary analysis of the Fap system revealed homologs in many additional bacterial genera and expanded the number of unique sequences in genera previously known to harbor the Fap systems considerably. However, although the NCBI RefSeq database has dramatically expanded since our first evolutionary study of the Fap system [17] and currently (8/1/2018) include genomic data for 48,788 bacterial species (https://www.ncbi.nlm.nih.gov/refseq/statistics/), these species represent only a fraction of the estimated microbial diversity on Earth, which has been proposed to range from millions to trillions of species [69]. The Fap system will probably be discovered in many additional genera in the future.

The ecological importance of the Fap system remains poorly understood, although increasing evidence indicates a role in pathogenesis and plant root colonization. It would, therefore, be relevant to investigate whether *P. aeruginosa* produces Fap fibrils when colonizing the lungs of patients suffering from cystic fibrosis or when causing chronic wounds. We have previously shown that Fap proteins can be detected and roughly quantified by label-free quantitative proteomic directly in complex samples that have been pretreated with concentrated formic acid [70]. This provides a simple approach to screen many samples for the presence of Fap amyloids. The spatial arrangement of the amyloids can subsequently be analyzed using Fap-specific fluorescent probes or antibodies in combination with confocal laser scanning microscopy [27]. A similar approach can be used to investigate the presence of Fap amyloid produced by PGPR upon interaction with plant roots. Another approach to learn more about the ecology of the Fap system would be to investigate how the system is regulated. This can be done by investigating how environmental factors, for example, substrate availability or presence of specific quorum sensing molecules, affect transcription of the *fap* operon or by the characterization of transcription factors that bind to the *fap* promoter [71], [72].

The structural understanding of curli components has advanced significantly since the structural determination of curli transporter CsgG [54], [56], [57], [73], [74]. The first glimpses into the Fap systems are now becoming available. The structure of the membrane-embedded domain of the Fap pore revealed a new mode of amyloid secretion that is distinct from curli [41], [45]. To understand the precise mechanistic differences, we first need to advance our atomistic structural insight into the whole Fap machinery, that is, all key components of the system and their complexes. Many of the key stages of Fap biogenesis are potential intervention points for inactivating the Fap pathway. A major aim will be to understand how the fiber component FapC is kept in a soluble monomeric form within the bacterial cell and delivered to an open FapF export channel.

An exciting opportunity for functional amyloids is their exploitation in bioengineering and material sciences [51], [52], [75], [76]. Their modular nature with a core amyloid fold and the potential to introduce new functionality into subunits, resulting fibers or higher-order 3D structures are particularly attractive features. As for other functional amyloid systems, Fap fibers are likely to be tolerant to the introduction of new functionalities while retaining the unique assembly and mechanical properties.

## Appendix A Supplementary data

Supplementary data 1 Image 1

Supplementary figures Image 2
