# Supplementary material for: Ecology and Biogenesis of Functional Amyloids in Pseudomonas
Source: J Mol Biol. 2018 Oct 12;430(20):3685–95. doi: 10.1016/j.jmb.2018.05.004 (PMC6173800; doi:10.1016/j.jmb.2018.05.004)
Supplement: Supplementary file 2 — Supplementary figures [file mmc2.docx]

Ecology and Biogenesis of Functional amyloids in *Pseudomonas*

Supplementary Information

**Authors:** Sarah L. Rouse^a^, Stephen J. Matthews^a^, and Morten S. Dueholm^b,c^

**Affiliation:**

^a^Department of Life Sciences, Imperial College London, South Kensington Campus, London, SW72AZ, UK.

^b^Center for Microbial Communities, Department of Chemistry and Bioscience, Aalborg University, Aalborg, Denmark.

^c^Correspondence to: Morten Simonsen Dueholm, Center for Microbial Communities, Department of Chemistry and Bioscience, Aalborg University, Fredrik Bajers Vej 7H, 9220 Aalborg, Denmark; Phone: +45 9940 3749; Fax: Not available; E-mail: md@bio.aau.dk

**Conflict of interest:**

The authors declare no conflict of interest.


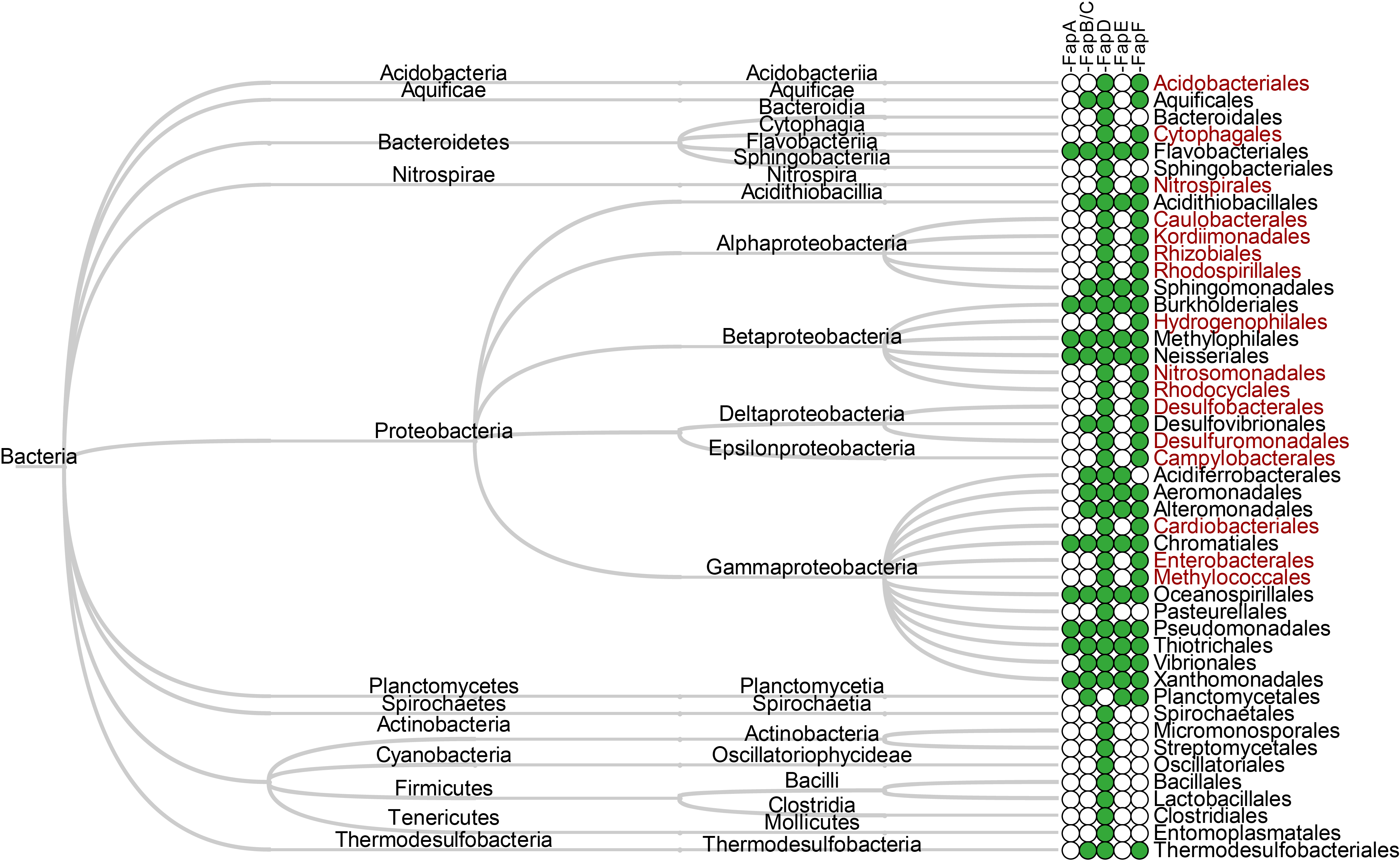


**Figure S1: Phylogenetic distribution of the Fap system.** Fap protein homologs were identified in the non-redundant bacterial RefSeq protein database v. 83 using the previously described profile hidden Markov models [1]. NCBI’s Batch Entrez was used to extract metadata for identical protein groups, and this information was used to identify genomes containing *fap*-gene homologs. Only proteins, which were encoded in the same genomic region as at least one other Fap protein (<5000 bp to nearest *fap*-gene neighbour) was included. This was done to remove false positive FapD and FapF hits. Note that some strains encoded several copies of FapD in the same genomic region. The taxonomic analysis was performed based on the NCBI taxonomy and visualised using MEGAN 6.0 [2]. The presence of individual Fap components in each order is illustrated in the filled circles left of the order names. Orders which encodes only FapD and FapF are highlighted with red text.


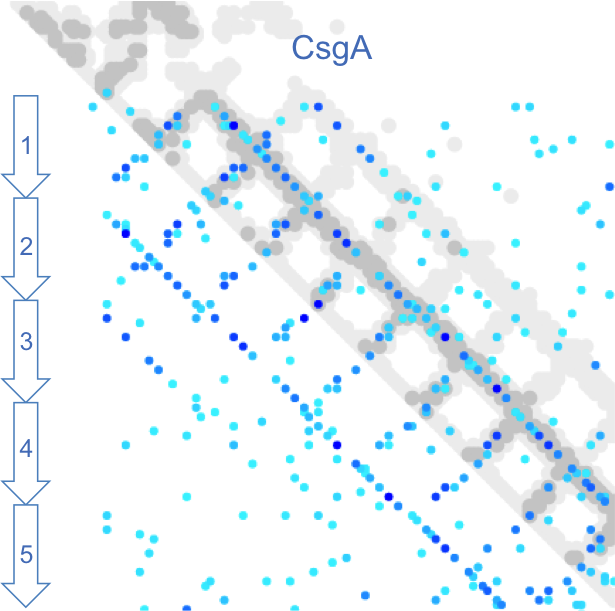


**Figure S2: Covariance data for Tian et al. CsgA model.** Expanded plots of Figure 5. The approximate repeat regions of CsgA are indicated by arrows. Grey shading corresponds to distances calculated from the CsgA model coordinate file.


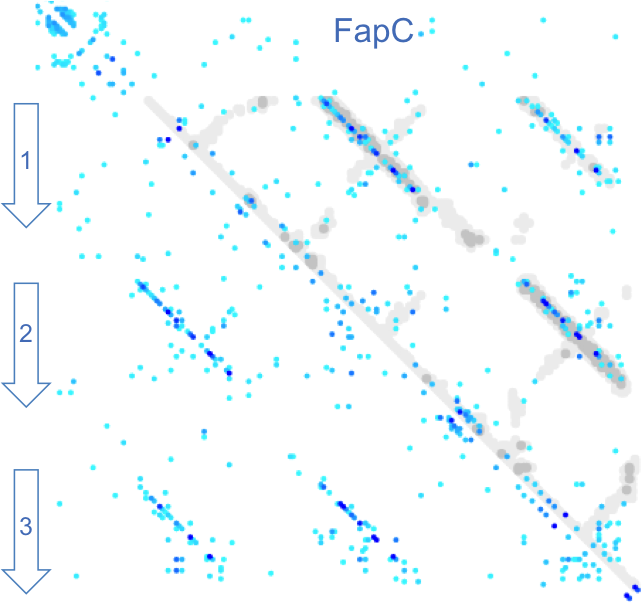


**Figure S3: Covariance data for FapC model.** Expanded plots of Figure 5. The approximate repeat regions of FapC are indicated by arrows. Grey shading corresponds to distances calculated from the FapC model coordinate file.


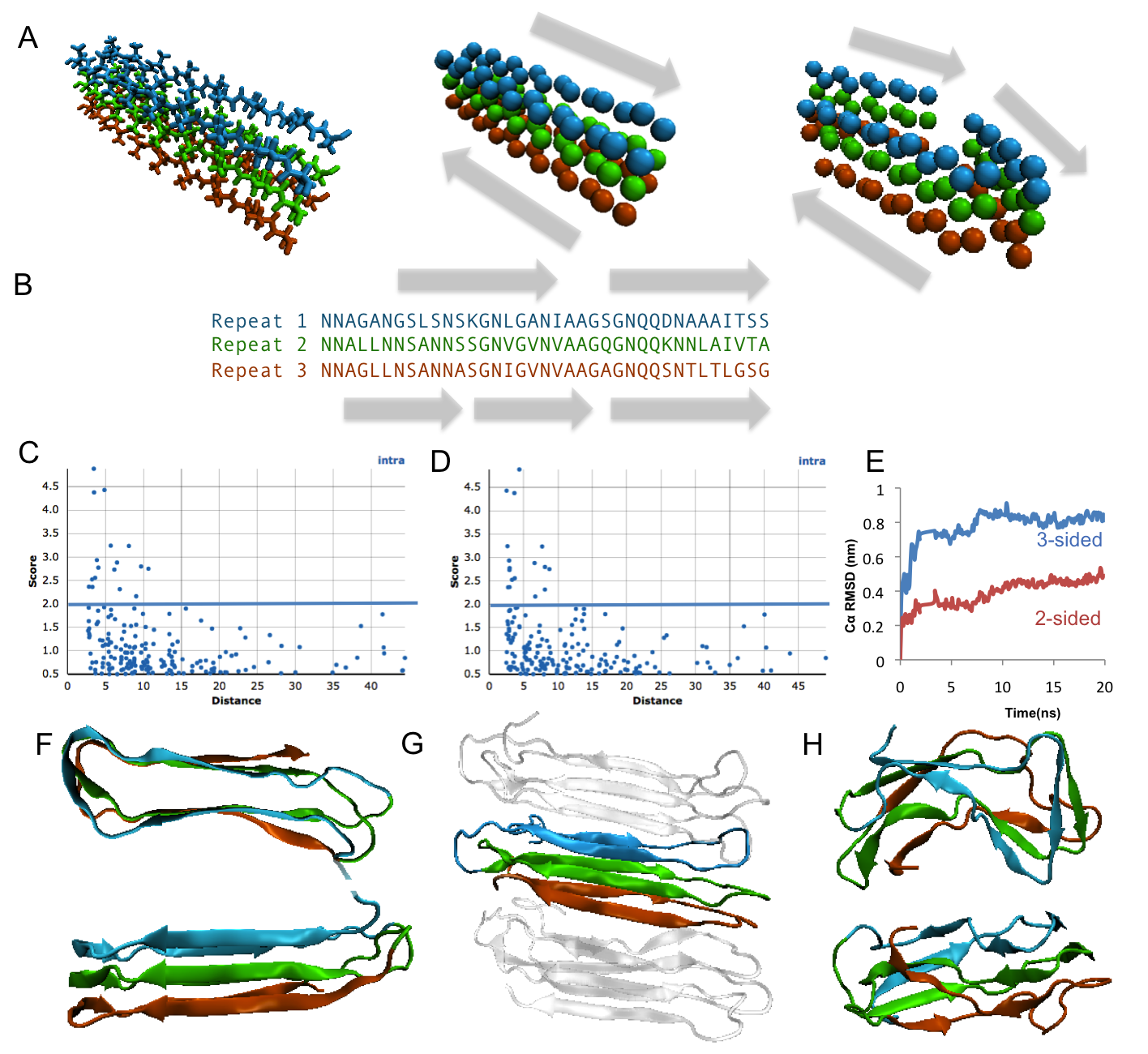


**Figure S4. Generating the FapC model.** Gremlin covariance analysis was used to identify the three repeat regions of 37 residues each and guide initial model generation. A). 3 sets of identical idealised polyalanine parallel beta strands were built in Pymol (Version 1.8 Schrödinger, LLC). Two simple models in which each repeat folds to either a 2-sided or 3-sided strand were used. B) Modeller was used to thread the FapC core repeat sequence as a single polypeptide chain onto this CA template, as indicated approximately by grey arrows on the sequence and template image. Each initial model of the fibre core was consistent with the covariance data, with 94% (2-sided, C) and 100% (3-sided, D) of the highest scoring (> 2 score from Gremlin) contacts below a distance cut off of 1 nm. E) Molecular simulations of the two sided monomer and a trimer generated by stacking 3 molecules (F) indicated the 2-sided models were stable over a 20 ns timescale, with snapshots of the protein after 20 ns shown. G) The 3-sided models were unstable and collapsed within a 20 ns timescale, snapshot at 10 ns shown. The final model of FapC UK4 including the disordered linker regions was generated using Modeller [3] by threading onto the core template. Simulations were performed in 0.15 M NaCl at 310 K using the gromacs (www.gromacs.org) simulation software with the GROMOS96 53a6 force field [4] as described by Rouse *et al.* [5].

**References**

[1] M.S. Dueholm, D. Otzen, P.H. Nielsen, Evolutionary insight into the functional amyloids of the pseudomonads, PLoS One. 8 (2013) e76630. doi:10.1371/journal.pone.0076630.

[2] S. Beier, R. Tappu, D.H. Huson, Functional Analysis in Metagenomics Using MEGAN 6, in: Funct. Metagenomics Tools Appl., Springer International Publishing, Cham, 2017: pp. 65–74. doi:10.1007/978-3-319-61510-3_4.

[3] A. Sali, Comparative protein modeling by satisfaction of spatial restraints, Mol. Med. Today. 1 (1995) 270–277. doi:10.1016/S1357-4310(95)91170-7.

[4] C. Oostenbrink, A. Villa, A.E. Mark, W.F. Van Gunsteren, A biomolecular force field based on the free enthalpy of hydration and solvation: The GROMOS force-field parameter sets 53A5 and 53A6, J. Comput. Chem. 25 (2004) 1656–1676. doi:10.1002/jcc.20090.

[5] S.L. Rouse, W.J. Hawthorne, J.-L. Berry, D.S. Chorev, S.A. Ionescu, S. Lambert, F. Stylianou, W. Ewert, U. Mackie, R.M.L. Morgan, D. Otzen, F.-A. Herbst, P.H. Nielsen, M. Dueholm, H. Bayley, C. V. Robinson, S. Hare, S. Matthews, A new class of hybrid secretion system is employed in Pseudomonas amyloid biogenesis, Nat. Commun. 8 (2017) 263. doi:10.1038/s41467-017-00361-6.
